# *Trichosporon inkin* meningitis in a pediatric patient diagnosed via metagenomic sequencing

**DOI:** 10.1128/asmcr.00053-25

**Published:** 2025-08-08

**Authors:** Kathryn Phillips, Karen P. Acker, Jin-Young Han, Christine M. Salvatore, Sallie R. Permar, Rebecca Marrero Rolón, Jamie Marino, Claire Dysart, David M. Berman, Charles Y. Chiu, Sarah E. Kidd, Lars F. Westblade, Melanie M. Dubois

**Affiliations:** 1 Department of Pediatrics, Weill Cornell Medicine 12295, New York, New York, USA; 2 Department of Pathology and Laboratory Medicine, Weill Cornell Medicine 12295, New York, New York, USA; 3 Karius, Inc, Redwood City, California, USA; 4 Department of Laboratory Medicine, University of California 8785, San Francisco, California, USA; 5 Department of Medicine, University of California San Francisco 8785, San Francisco, California, USA; 6 National Mycology Reference Centre, SA Pathology 10107, Adelaide, Australia; 7 Faculty of Sciences, Engineering and Technology, University of Adelaide 1066, Adelaide, Australia; Vanderbilt University Medical Center, Nashville, Tennessee, USA

**Keywords:** *Trichosporon inkin*, meningitis, pediatric, metagenomic next-generation sequencing

## Abstract

**Background:**

*Trichosporon* has emerged as an important cause of invasive fungal infections in immunocompromised patients. There are limited data on invasive *Trichosporon* infections in children.

**Case Summary:**

We report a case of culture-negative *Trichosporon inkin* meningitis diagnosed via metagenomic next-generation sequencing of plasma and cerebrospinal fluid in an infant with retinoblastoma. In addition, we highlight the role of β-1,3-D-glucan in the diagnosis and therapeutic monitoring of trichosporonosis, and cross-reactivity of the cryptococcal antigen lateral flow assay with *T. inkin*.

**Conclusion:**

This diagnosis, which was challenging to make in the absence of a positive culture, highlights the utility of metagenomic sequencing methods and fungal biomarkers in identifying infectious agents and ensuring timely diagnosis and management of patients with rare fungal infections of the central nervous system.

## INTRODUCTION

*Trichosporon* is a yeast-like fungus found in the environment and a component of the skin and gastrointestinal tract microbiota of humans (1–3). *Trichosporon* has emerged as an important cause of infection in immunocompromised patients, including those with neutropenia and hematological malignancies (1). *Trichosporon asahii* is the most frequent cause of trichosporonosis and has been implicated in fungemia, meningitis, pneumonia, endocarditis, abscesses, and other serious infections (1, 4). Another species, *Trichosporon inkin,* is associated with the benign hair condition white piedra, but is also known to cause invasive infection (1, 2). Invasive trichosporonosis is associated with a mortality rate as high as 80%, particularly in the setting of delayed diagnosis (1).

To date, there are few reports of *T. inkin* invasive infections in humans and only one report of *T. inkin* meningitis in an adult (2). We present a case of an immunocompromised child with trilateral retinoblastoma on chemotherapy who developed a fungal infection of the central nervous system (FI-CNS) with *Trichosporon* meningitis, in which the presence of *T. inkin* was identified through metagenomic next-generation sequencing (mNGS) of plasma and cerebrospinal fluid (CSF).

## CASE PRESENTATION

An 11-month-old male with trilateral retinoblastoma diagnosed at 8 months of age was admitted to a tertiary-care cancer center for febrile neutropenia. At the time of his retinoblastoma diagnosis, he underwent right frontal endoscopic diagnostic biopsy of a pineal mass and received local chemotherapy with topotecan injections followed by three cycles of systemic chemotherapy, with the last cycle completed 5 days prior to admission. Of note, he had a history of methicillin-susceptible *Staphylococcus aureus* (MSSA) bacteremia at 8 months of age.

**Fig 1:**
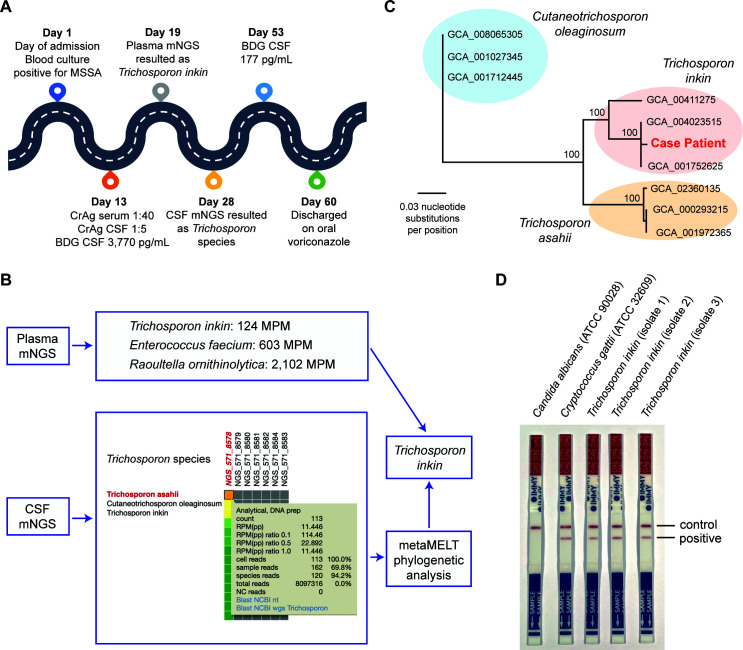
Hospitalization course and laboratory testing for the case patient. (**A**) Timeline of microbiologic testing. (**B**) Results of clinical mNGS testing for the patient. Plasma microbial cell-free DNA mNGS identified three different organisms, of which one was *Trichosporon inkin*. CSF mNGS identified the presence of *Trichosporon* species, which was later determined to be *T. inkin* by metaMELT phylogenetic analysis. (**C**) Phylogenetic analysis of concatenated *Trichosporon* species reads recovered from the patient’s CSF specimen using the metaMELT algorithm. Briefly, the *Trichosporon* species reads were mapped to their corresponding regions extracted from nine fungal reference genomes. Maximum likelihood phylogenetic analysis of the concatenated patient reads in parallel with the concatenated corresponding regions extracted from the nine fungal genomes identified the most likely species as *T. inkin*. Each fungal genome is denoted by a GenBank accession number. (**D**) *T. inkin* cross-reactivity with the cryptococcal antigen lateral flow assay (IMMY) with clinical isolates recovered from three unique patients. mNGS, metagenomic next-generation sequencing; CSF, cerebrospinal fluid; MPM, molecules per microliter; metaMELT, metagenomic multiple extended locus typing.

Prior to transfer, the patient was started on piperacillin-tazobactam (90 mg/kg/dose Q6) and vancomycin (15 mg/kg/dose Q6). Blood cultures were positive for MSSA (Fig. 1A), which was attributed to a central line infection. Blood cultures cleared within 2 days, and after 4 days on antimicrobial treatment, the patient defervesced and was transitioned to oxacillin (35 mg/kg/dose Q4) monotherapy. He subsequently underwent central line removal, and shortly after, redeveloped fever with mental status changes. Antimicrobials were broadened to cefepime (50 mg/kg/dose Q8), vancomycin (15 mg/kg/dose Q6), and acyclovir (10 mg/kg/dose Q8) due to clinical concern for meningitis. Lumbar puncture was deferred given clinical instability. Brain magnetic resonance imaging (MRI) showed diffuse areas of subacute infarcts involving the basal ganglia, thalamus, midbrain, and hippocampi in addition to multiple mural enhancing aneurysms throughout the anterior cerebral circulation, concerning for mycotic aneurysms, also known as infectious intracranial aneurysms (5).

The patient was subsequently transferred to our hospital for coil embolization of a 7 mm × 11 mm mycotic aneurysm of the right anterior cerebral artery. Repeat MRI on postoperative day 3 showed new leptomeningeal enhancement with basilar predominance and new mycotic aneurysms present in the posterior cerebral circulation. Blood culture (bacterial) and CSF cultures (bacterial, fungal, and mycobacterial) were obtained, and antimicrobials were broadened to meropenem (40 mg/kg/dose Q8), liposomal amphotericin B (5 mg/kg/dose Q24), along with rifampin (15 mg/kg/dose Q24), isoniazid (10 mg/kg/dose Q24), pyrazinamide (35 mg/kg/dose Q24), and ethambutol (20 mg/kg/dose Q24) for empiric *Mycobacterium tuberculosis* (MTB) disease treatment.

CSF testing revealed a β-1,3-D-glucan (BDG) (Associates of Cape Cod, Inc., Falmouth, MA) level of 3,770 pg/mL, positive cryptococcal antigen (CrAg) (titer 1:5) (IMMY, Norman, OK), and negative bacterial cultures. CSF fungal and mycobacterial cultures were held for 4 weeks and 8 weeks, respectively, and reported as negative. Serum testing revealed elevated BDG (211 pg/mL), positive CrAg (titer 1:40), and negative *Aspergillus* galactomannan (Bio-Rad Laboratories, Inc., Hercules, CA). Blood cultures were negative. QuantiFERON-TB Gold Plus testing (Qiagen, Hilden, Germany) was indeterminate. Elevated BDG levels in serum and CSF raised concern for fungal meningitis. In the setting of a positive CrAg result, fluconazole (12 mg/kg/dose Q24) was added to amphotericin B. MTB CSF PCR testing was negative, and anti-MTB therapy was discontinued.

Given the unclear etiology of meningitis, mNGS testing of plasma (Karius, Redwood City, CA) and CSF (University of California San Francisco, San Francisco, CA) were performed (Fig. 1B). Within 5 days, plasma microbial cell-free DNA sequencing reported detection of *T. inkin* 124 molecules per microliter (MPM), *Enterococcus faecium* 603 MPM, and *Raoultella ornithinolytica* 2,102 MPM. *T. inkin* was thought to be more consistent with his clinical presentation, and the additional organisms were presumed to be colonizers. Fifteen days after collection, CSF mNGS testing reported the detection of a *Trichosporon* species, confirming a diagnosis of *Trichosporon* meningitis in this patient. The top species-level hits, ranked in descending order by number of reads, were *T. asahii, Cutaneotrichosporon oleaginosum,* and *T. inkin*. Further evaluation using a novel analytic method, metagenomic multiple extended locus typing (metaMELT), identified the detected species as most likely *T. inkin* based on phylogenetic analysis of the patient’s strain compared to nine fungal reference genomes (Fig. 1C) (6). The antimicrobial regimen was narrowed to voriconazole (9 mg/kg/dose Q12 for 2 doses, followed by 8 mg/kg/dose Q12) for *Trichosporon* meningitis and nafcillin (50 mg/kg/dose Q6) for MSSA bacteremia.

Despite appropriate antifungal therapy, the patient developed intraventricular hemorrhage and hydrocephalus, likely from aneurysm rupture, requiring external ventricular drain placement and eventually ventriculoperitoneal shunt placement. While subsequent MRI studies demonstrated improvement of leptomeningeal enhancement, the patient continued to develop new punctate infarcts and mycotic aneurysms in both the anterior and posterior cerebral circulations. Once on appropriate antifungal therapy, dexamethasone 0.15 mg/kg/dose daily was started to reduce central nervous system inflammation, then increased to 0.15 mg/kg/dose every 6 hours for 1 week, given persistent vasospasms, followed by a taper. CSF BDG levels decreased while on antifungal therapy, to 338 pg/mL 1 week after and 177 pg/mL 5 weeks after antifungal therapy was initiated. He was discharged home on oral voriconazole (11 mg/kg/dose Q12) and demonstrated significant neurologic improvement. Six months after his initial diagnosis, CSF mNGS and CSF BDG were both negative.

## DISCUSSION

To our knowledge, this is the second reported case of *T. inkin* meningitis (2) and the first in a pediatric patient. There were many diagnostic challenges with this case. Initially, clinical concern was highest for a bacterial source of meningitis due to MSSA, given its association with mycotic aneurysms and his recent bacteremia. As his illness progressed and imaging demonstrated intracranial mycotic aneurysms and basilar meningitis, atypical causes were further investigated. The positive CrAg in both serum and CSF was concerning for cryptococcal meningitis, which is a common cause of meningitis in immunocompromised patients (7). The patient had elevated serum and CSF BDG, which is generally not considered to be produced by *Cryptococcus* species, although there have been reports of elevated BDG levels in immunocompromised patients with cryptococcal meningitis (8). The diagnosis was ultimately made through detection of *T. inkin* DNA in the plasma and CSF by mNGS. We suspect that the most likely source of infection was from gut translocation in the setting of neutropenia, as gastrointestinal colonization is common (1). Although a rare cause of meningitis in immunocompromised patients, *Trichosporon* fits with the characteristics of his illness, including basilar meningitis and the elevated BDG.

Cryptococcal immunoassays have demonstrated cross-reactivity with *T. asahii*, but have not been extensively tested with other *Trichosporon* species or related yeasts (7). Accordingly, we tested 0.5 McFarland suspensions of *T. inkin* isolates recovered from three unrelated patients with the IMMY CrAg assay; all three isolates yielded a positive reaction (Fig. 1D). The cross-reactivity observed with the IMMY CrAg lateral flow assay and *T. inkin* is a new preliminary finding and warrants further investigation of cryptococcal immunoassays with a wide range of basidiomycetous yeasts.

Another uniquely challenging aspect of this patient’s case was the presence of intracranial mycotic aneurysms, which are pseudoaneurysms that develop in response to infection, leading to inflammation and tissue destruction in multiple layers of the arterial wall (5). Pediatric mycotic aneurysms are most commonly associated with *S. aureus* and viridans group streptococci, with *Candida albicans* and *Aspergillus* species as the most commonly identified fungal causes (5). There are no reports of *T. inkin* associated with mycotic aneurysms, but there are reports caused by other *Trichosporon* species, including one in which *T. asahii* was identified as the cause of abdominal aorta and common iliac mycotic aneurysms (9). In another report, *Trichosporon* (identified only to the genus level) was noted as the cause of a posterior cerebral artery mycotic aneurysm (10). While we cannot confirm without histopathologic diagnosis that *T. inkin* was the definitive cause of mycotic aneurysms in our patient, we suspect that this is a possible explanation given the organism’s known pathogenic potential in immunocompromised hosts.

In our patient, we were uniquely able to diagnose *Trichosporon* meningitis through detection of *Trichosporon* DNA in both plasma and CSF using mNGS. mNGS of microbial DNA in plasma and CSF specimens has been used to expedite the broad-spectrum diagnosis of infectious etiologies, including fungal infections (11, 12). CSF BDG was also helpful for diagnosis and therapeutic monitoring of FI-CNS, as has been reported previously in pediatric patients (13). As fungal cultures remained negative, this case demonstrates the importance of mNGS testing as a diagnostic tool to ensure early diagnosis, appropriate targeted treatment, and optimized outcomes for patients with FI-CNS.

In conclusion, this case highlights the use of mNGS for timely diagnosis and treatment for patients with rare FI-CNS, including *T. inkin*, the role of BDG in the diagnosis and therapeutic monitoring of trichosporonosis, and cross-reactivity of the IMMY CrAg lateral flow assay with *T. inkin*.

## References

[1] Mehta V, Nayyar C, Gulati N, Singla N, Rai S, Chandar J. 2021. A comprehensive review of Trichosporon spp.: an invasive and emerging fungus. Cureus 13:e17345. doi:10.7759/cureus.17345 34567886 PMC8451254

[2] Milan EP, Silva-Rocha WP, de Almeida JJS, Fernandes TUG, de Araújo Prudente AL, de Azevedo MF, Francisco EC, de Azevedo Melo AS, Colombo AL, Chaves GM. 2018. Trichosporon inkin meningitis in Northeast Brazil: first case report and review of the literature. BMC Infect Dis 18:470. doi:10.1186/s12879-018-3363-7 30227852 PMC6145100

[3] Westblade LF, Burd EM, Lockhart SR, et al.. 2023. Larone’s medically important fungi: a guide to identification. 7th edition. Wiley-ASM Press.

[4] Foster CE, Edwards MS, Brackett J, Schady DA, Healy CM, Baker CJ. 2018. Trichosporonosis in pediatric patients with a hematologic disorder. J Pediatric Infect Dis Soc 7:199–204. doi:10.1093/jpids/pix031 28510690

[5] Flores BC, Patel AR, Braga BP, Weprin BE, Batjer HH. 2016. Management of infectious intracranial aneurysms in the pediatric population. Childs Nerv Syst 32:1205–1217. doi:10.1007/s00381-016-3101-7 27179531

[6] Chiu CY, Servellita V, de Lorenzi-Tognon M, Benoit P, Sumimoto N, Foresythe A, Cerqueira FM, Williams-Bouyer N, Ren P, Herrera LNS, Gaston DC, Sayyad L, Whitmer SL, Klena J, Vikram HR, Gold JAW, Gade L, Parnell L, Misas E, Chiller TM, Griffin IS, Basavaraju SV, Smith DJ, Litvintseva AP, Chow NA. 2025. Metagenomic identification of Fusarium solani strain as cause of US fungal meningitis outbreak associated with surgical procedures in Mexico, 2023. Emerg Infect Dis 31:948–957. doi:10.3201/eid3105.241657 40180580 PMC12044249

[7] Liu Y, Kang M, Wu S-Y, Wu L-J, He L, Xiao Y-L, Zhang W-L, Liao Q-F, Deng J, Chen Z-X, Ma Y. 2022. Evaluation of a Cryptococcus capsular polysaccharide detection FungiXpert LFA (lateral flow assay) for the rapid diagnosis of Cryptococcosis. Med Mycol 60:myac020. doi:10.1093/mmy/myac020 35362524

[8] Rhein J, Boulware DR, Bahr NC. 2015. 1,3-β-D-glucan in cryptococcal meningitis. Lancet Infect Dis 15:1136–1137. doi:10.1016/S1473-3099(15)00306-0 26461948

[9] Quintana JL, Carreras X, Salcedo AS, Ruíz LT, Gonzales MM, Del Castillo Mory A, Cheng HC, Gonzalez AC. 2023. Mycotic aneurysms due to Trichosporon asahii in a patient with ulcerative colitis under immunosuppression. IDCases 32:e01794. doi:10.1016/j.idcr.2023.e01794 37214183 PMC10196954

[10] Shibao S, Kaburaki M, Saito K, Tomita H. 2022. A case of subarachnoid and intracerebral hemorrhages complicated by trichosporonosis. Surg Neurol Int 13:472. doi:10.25259/SNI_780_2022 36324937 PMC9610440

[11] Blauwkamp TA, Thair S, Rosen MJ, Blair L, Lindner MS, Vilfan ID, Kawli T, Christians FC, Venkatasubrahmanyam S, Wall GD, Cheung A, Rogers ZN, Meshulam-Simon G, Huijse L, Balakrishnan S, Quinn JV, Hollemon D, Hong DK, Vaughn ML, Kertesz M, Bercovici S, Wilber JC, Yang S. 2019. Analytical and clinical validation of a microbial cell-free DNA sequencing test for infectious disease. Nat Microbiol 4:663–674. doi:10.1038/s41564-018-0349-6 30742071

[12] Benoit P, Brazer N, de Lorenzi-Tognon M, Kelly E, Servellita V, Oseguera M, Nguyen J, Tang J, Omura C, Streithorst J, Hillberg M, Ingebrigtsen D, Zorn K, Wilson MR, Blicharz T, Wong AP, O’Donovan B, Murray B, Miller S, Chiu CY. 2024. Seven-year performance of a clinical metagenomic next-generation sequencing test for diagnosis of central nervous system infections. Nat Med 30:3522–3533. doi:10.1038/s41591-024-03275-1 39533109 PMC11645279

[13] Salvatore CM, Chen TK, Toussi SS, DeLaMora P, Petraitiene R, Finkelman MA, Walsh TJ. 2016. (1→3)-β-d-Glucan in cerebrospinal fluid as a biomarker for Candida and Aspergillus infections of the central nervous system in pediatric patients . J Ped Infect Dis 5:277–286. doi:10.1093/jpids/piv014 PMC6281134 26407252

